# *Erigeron breviscapus* (Vant.) Hand-Mazz.: A Promising Natural Neuroprotective Agent for Alzheimer’s Disease

**DOI:** 10.3389/fphar.2022.877872

**Published:** 2022-04-26

**Authors:** Xiaoyu Dong, Shengtao Qu

**Affiliations:** ^1^ Department of Neurology, Shengjing Hospital of China Medical University, Shenyang, China; ^2^ Department of Neurosurgery, Shengjing Hospital of China Medical University, Shenyang, China

**Keywords:** Alzheimer’s disease, traditional Chinese medicine, *Erigeron breviscapus* (vant.) hand-mazz., pathogenesis, treatment

## Abstract

Alzheimer’s disease (AD) is the most common neurodegenerative disease and is characterized by progressive cognitive dysfunction and memory loss in the elderly, which seriously affects the quality of their lives. Currently, the pathogenesis of AD remains unclear. Molecular biologists have proposed a variety of hypotheses, including the amyloid-β hypothesis, tau hyperphosphorylation hypothesis, cholinergic neuron injury, inflammation caused by an abnormal immune response, and gene mutation. Drugs based on these pathological studies, including cholinesterase inhibitors and N-methyl-D-aspartate receptor antagonists, have achieved a certain level of efficacy but are far from meeting clinical needs. In the recent years, some important advances have been made in the traditional Chinese medicine treatment of AD. *Erigeron breviscapus* (Vant.) Hand-Mazz. (EBHM) is an important medicinal plant distributed in Yunnan Province, China. Studies have shown that EBHM and its active ingredients have a variety of pharmacological effects with good therapeutic effects and wide application prospects for cognitive disability-related diseases. However, to our best knowledge, only few review articles have been published on the anti-AD effects of EBHM. Through a literature review, we identified the possible pathogenesis of AD, discussed the cultivation and phytochemistry of EBHM, and summarized the pharmacological mechanism of EBHM and its active ingredients in the treatment of AD to provide suggestions regarding anti-AD therapy as well as a broader insight into the therapeutic potential of EBHM.

## Introduction

Alzheimer’s disease (AD) is a chronic degenerative disease of the central nervous system that mainly affects the elderly (Landry and Liu-Ambrose, 2014). It was first described in 1906 by Alois Alzheimer, a German doctor. The main clinical features are progressive cognitive decline, language impairment, and mental and behavioral abnormalities (Murray, 2013). According to statistics from Alzheimer’s Disease International (ADI), there were approximately 46.8 million patients with dementia worldwide in 2015—which is expected to increase to 131.5 million by 2050—among which patients with AD account for 50%–70% (Mason, 2015). At present, there are approximately six million patients with AD in China, and the incidence rate of AD in women is higher than that in men. The total social and economic costs long-term care and hospital services for AD is about 167.7 billion US dollars, causing huge economic and social family pressure (Jia et al., 2014; Jia et al., 2018). At the neuropathological level, AD is characterized by senile plaques (SPs) formed by the aggregation of beta-amyloid (Aβ) and neurofibrillary tangles (NFTs) formed by the aggregation of abnormally phosphorylated tau protein. Although the pathogenesis of AD has not been completely clarified, gene mutations, cholinergic injury, immune inflammatory mechanisms, oxidative stress, and mitochondrial damage may be involved (Jun et al., 2014; Day et al., 2016; Howard et al., 2020). Currently, there is a lack of specific drugs for the treatment of AD in clinical practice, most of which are aimed at improving clinical symptoms and delaying disease progression. Commonly used drugs include cholinesterase inhibitors, non-competitive N-methyl-D-aspartic acid (NMDA) receptor antagonists, and drugs to improve brain metabolism (Joe and Ringman, 2019; Tolar et al., 2020). However, the clinical efficacy of these drugs is not ideal and does not meet the needs of clinical treatment.

As an important part of global medicine, traditional Chinese medicine (TCM) plays an irreplaceable role in the treatment of AD. In the recent years, an increasing number of scholars have performed in-depth studies and have provided theoretical support regarding the therapeutic potential of TCM for AD (Howes et al., 2017; Lu et al., 2021). For example, 6-shogaol, a bioactive component of ginger, may reduce memory impairment by inhibiting glial cell activation in animal models of dementia (Moon et al., 2014). δ-9-tetrahydrocannabinol and cannabidiol significantly reduce the level of soluble Aβ42, inhibit neurotoxicity and inflammatory factor expression, and improve memory in APP/PS1 transgenic AD mice (Aso et al., 2015). *E. breviscapus* (Vant.) Hand-Mazz. (EBHM) is a perennial herbaceous plant of the genus Euphorbia in the Asteraceae family, which is usually distributed in grassy and open forests on sunny slopes at altitudes of 1,700–3,000 m in Yunnan Province. EBHM and its active ingredients (such as baicalin and scutellarin) have a variety of pharmacological effects, including improved blood circulation, an anti-inflammatory response, an anti-oxidative stress response, and inhibition of apoptosis (Fan et al., 2021). However, the anti-AD mechanism of EBHM and its active ingredients have not been clearly elucidated. Here, we review the results of EBHM in the treatment of AD and discuss the phytochemistry and cultivation of EBHM to further elaborate on the pharmacological mechanism of EBHM in the treatment of AD. This provides suggestions for the development of new therapeutic strategies for AD and may play a role in promoting the clinical application of EBHM.

## *Erigeron breviscapus* (Vant.) Hand-Mazz

**FIGURE 1:**
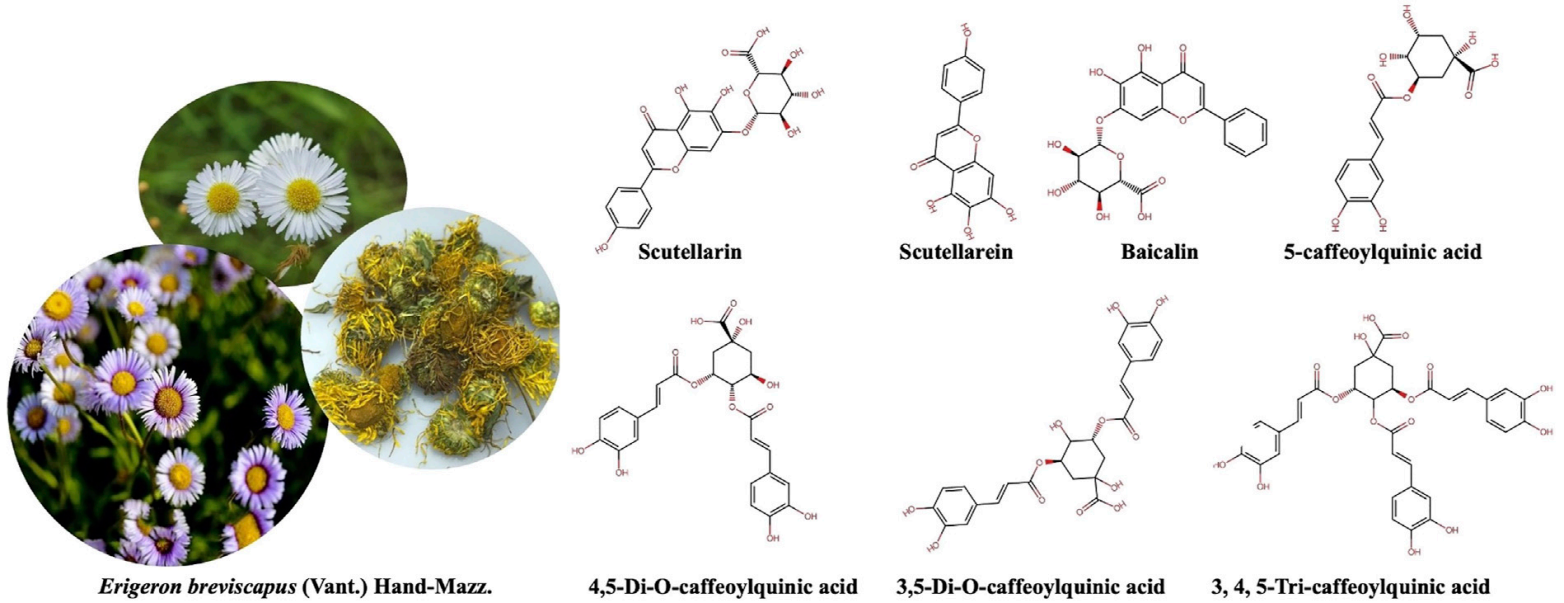
The flower and main chemical structures of *Erigeron breviscapus* (Vant.) Hand-Mazz.

EBHM, also known as *Dengzhan Asarum*, was first recorded in South Yunnan Materia Medica. It belongs to the short pavilion fleabane group and is classified as a fleabane in Asteraceae family. There are more than 200 species of fleabane, but only three species are medicinal. EBHM is a perennial herbaceous plant that flowers year-round. Traditionally, whole grass is used as medicine and is collected in the summer and autumn. According to data records, EBHM is mainly distributed in China’s Hunan, Guangdong, Guangxi, Guizhou, and Sichuan provinces and grows at an altitude of 1,700–3,000 m of open hillside grassland and forest margins. EBHM accounts for more than 95% of the total resources in China and is becoming the main source of natural medicinal material in Yunnan Province. The therapeutic properties of EBHM include detoxification (Mo et al., 2018), blood circulation activation (Liu et al., 2021), channel and collateral activation (Shi et al., 2015), inflammation reduction (Zhu et al., 2018), and pain relief (Wu et al., 2021). A variety of dosage forms, such as tablets, capsules, oral liquids, and injections, have been developed based on the different doses and delivery routes of EBHM extracts. EBHM extracts have been used to treat a variety of common diseases, such as cerebral infarction and coronary heart disease, and have a large market share in China (Figure 1) (Fan et al., 2021).

## *Erigeron breviscapus* (Vant.) Hand-Mazz. Cultivation

Owing to years of blind mining, wild EBHM resources are increasingly being reduced and destroyed, and at present, artificial propagation of EBHM is mainly adopted. EBHM has wide adaptability and can maintain normal growth and development at 6°C–25°C. EBHM growth requires adequate water. The effect of water on the EBHM seedling stage is significant, and the survival of seedlings after embryo germination is directly dependent on environmental conditions owing to their smaller seed size. Adult EBHM plants develop root systems that are highly adaptable to harsh environments. In dry seasons, EBHM plants have slow vegetative growth and thickened leaf matter, whereas during rainy seasons, they grow rapidly. EBHM is a heliophyte that requires large amounts of light. Wild EBHM plants are mainly distributed in open hillsides that faces the Sun, while artificially cultivated EBHM plants mainly grow under plastic film or in greenhouses due to difficulties in seedling propagation, insufficient light, weak growth, and vulnerability to disease infection. However, under artificial control and management, the emergence rate of EBHM may be greatly improved, and the production cost is low. EBHM field production provides a large number of seedlings, which is currently an ideal method of seedling propagation.

## Phytochemistry of *Erigeron breviscapus* (Vant.) Hand-Mazz

**FIGURE 2:**
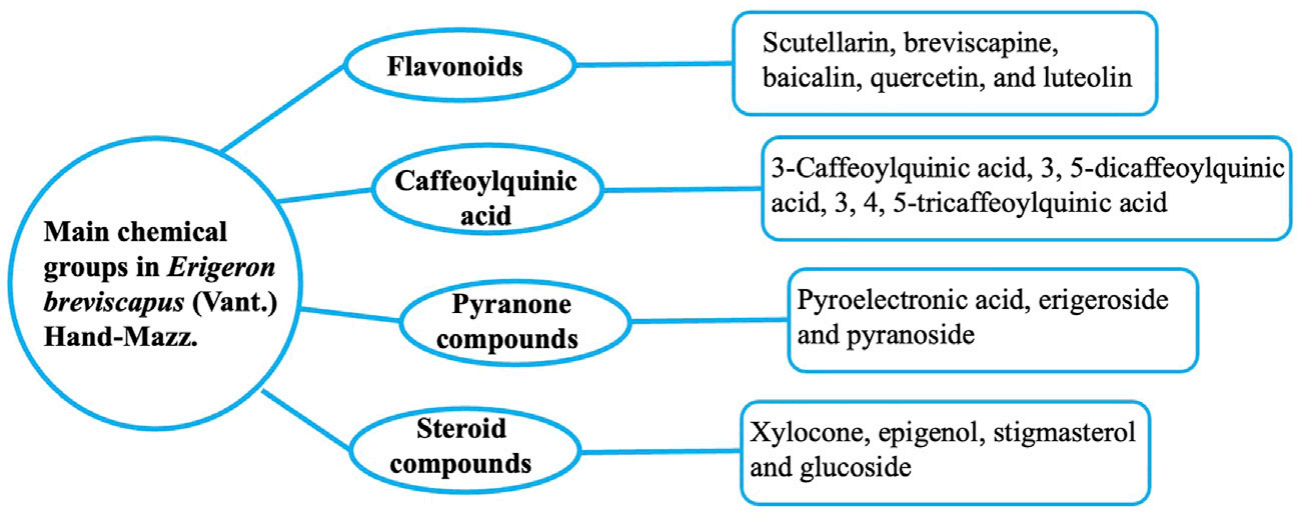
Main chemical classes present in *Erigeron breviscapus* (Vant.) Hand-Mazz.

The chemical components of EBHM include flavonoids, caffeoylquinic acid (CQA), and pyranones (Figure 2). Flavonoids and CQAs are considered the main active ingredients of EBHM due to their therapeutic effects. Flavonoids and glycosides have been isolated from EBHM (accounting for approximately 47.41% of all reported EBHM compounds), including scutellarin, breviscapine, baicalin, quercetin, and luteolin (Qu et al., 2001; Xia et al., 2007; Tao et al., 2008; Tian et al., 2017). CQA consists of caffeic acid, 3-O-caffeoylquinic acid (3-CQA), dicaffeoylquinic acid (3, 5-dicaffeoylquinic acid, 3, 5-di-CQA), and tricaffeoylquinic acid (3, 4, 5-tricaffeoylquinic acid, 3, 4, 5-tri-CQA), which account for 29.7% of all the reported EBHM compounds (Jiang et al., 2017). In addition, EBHM also includes pyranone compounds, such as pyroelectronic acid, erigeroside, and pyranoside. Several steroid compounds, including epigenol, stigmasterol, and glucoside have also been found in EBHM. Subsequently, various components contained in EBHM have been discovered. The chemical constituents of EBHM mainly focus on the ethyl acetate portion of the ethanol extract and include 3-hydroxy-baicalin, 3-hydroxy-7-methoxy baicalin, 5,7,4’-trihydroflavone, cinnamic acid, methyl coffeate, p-methoxy cinnamic acid, (1R, 3R)-dihydroxyl-(4S, 5R)-methyl dicafeoxy cyclohexanate, methyl 1, 4-dihydroxy (3R, 5R)-dicafeoxy cyclohexanate, 3, 4-dihydroxy benzoic acid, p-hydroxybenzoic acid, quercetin-3-O-β-d-glucoside, 5, 7-dihydroxy chromogenic one, 3-O-coffeoyl-γ-quinone, and naringin. However, most flavonoids are converted by gut microbes. In addition, phenolic acids are widely metabolized in the body and their products vary. Owing to the limitations of experimental conditions and analytical methods, many metabolites are still unknown. Therefore, further studies of EBHM metabolites are required.

## *Erigeron breviscapus* (Vant.) Hand-Mazz. in Traditional Medicine

EBHM is an herbal TCM that has been used to treat cardiovascular and nervous system diseases for more than 600 years. Several case-control and randomized controlled studies have shown that EBHM has a good therapeutic effect on stable and unstable angina, acute myocardial infarction, essential hypertension, atrial fibrillation, heart failure, pulmonary heart disease, and acute hypertensive cerebral hemorrhage (Lu et al., 2013; Wang et al., 2015; Gao et al., 2017; Wang et al., 2020; Jia et al., 2021). In a randomized, double-blind, case-control study, Zhong et al. (2010) demonstrated that EBHM can improve glaucoma-related visual field defect symptoms.

EBHM has also been widely used clinically as the main medicinal ingredient in some Chinese medicine prescriptions. The Dengzhan Xixin injection (DZXX) and Dengzhan Shengmai (DZSM) capsules are the most widely used. Both TCMs can improve neurological function and quality of life and reduce the recurrence rate of patients with ischemic stroke (Yang et al., 2018; Cai et al., 2019). DZXX is a preparation of extracts from TCM EBHM and has been widely used in clinical treatment of cerebral ischemia sequelae in China for a long history. DZXX mainly contains scutellarin, 3,4-di-CQA, 3, 5-di-CQA, 4,5-di-CQA, caffeic acid and 5-CQA (Wang et al., 2015). In middle cerebral artery occlusion (MCAO) model of rats, DZXX administration memorably ameliorated pathological changes and neuronal loss, meanwhile, DZXX reduced the surged reactive oxygen species (ROS) and malondialdehyde (MDA), while increased the level of superoxide dismutase (SOD) (Yang et al., 2022). In addition, DZXX has been shown to improve the Barthel index and neurological deficit scores without adverse events in patients with acute ischemic stroke (Li et al., 2017). DZSM as a well-known TCM formula, is mainly comprised of EBHM, and supplemented with *Panax ginseng* C.A.Mey., *Ophiopogon japonicus* (Linn. f.) Ker-Gawl. and *Schisandra chinensis* (Turcz.) Baill., with functions of supplementing Qi and nourishing Yin, promoting blood circulation and strengthening brain (Mu et al., 2019). Recent studies have shown that the DZSM capsules and its main active ingredient scutellarin can protect neurons from ischemic injury *via* anti-inflammatory, anti-apoptotic and antioxidant activities (Wang et al., 2016; Sun et al., 2018). Moreover, DZSM capsules combined with donepezil hydrochloride have been reported to improve cognitive function and living ability in patients with AD and reduce the production of the neurotoxic substances nitric oxide (NO) and endothelin (ET) (Huang et al., 2021). In patients with vascular dementia, DZSM capsules combined with butylphthalide can effectively reduce vascular endothelial function injury, inhibit the oxidative stress response, and reduce the endoplasmic reticulum stress response, thereby reducing dementia symptoms and improving cognitive function (Ma and Sun, 2020). However, there is a need for high-quality, large-sample, randomized clinical trials to further validate these conclusions.

## Clinical Impact of *Erigeron breviscapus* (Vant.) Hand-Mazz

To further assess the clinical effect of EBHM, we searched the clinical trial website http://clinicaltrial.gov/ with keywords “*E*RIGERON *BREVISCAPUS* (VANT.) HAND-MAZZ.” and “Dengzhan” on 31 December 2021. The results showed that there were four clinical trials on EBHM. The first clinical trial applied *E. breviscapus* injection to evaluate TCM syndrome differentiation and the prognosis of acute ischemic stroke. In total, 500 patients with acute ischemic stroke were included in this study. After a 16-month follow-up, the results suggested that and *E. breviscapus* injection can improve the degree of disability and activities of daily living without definite adverse events in patients with acute ischemic stroke (Huang and Guo, 2010). The second clinical trial evaluated DZSM capsules for the TCM-integrated treatment program and efficacy in patients with ischemic stroke, in which 3,143 patients were included from 84 treatment centers. The study lasted for 48 months, and the results showed that DZSM capsules have good efficacy in patients with acute ischemic stroke. The third clinical trial evaluated the efficacy of DZSM capsules in the comprehensive treatment for the secondary prevention of ischemic stroke by TCM. The study involved 12,000 participants and lasted 24 months, and the results showed that DZSM capsules may reduce the recurrence rate of ischemic stroke within 1 year. The fourth clinical trial was regarding post-marketing safety monitoring of breviscapine powder injection, a registered study that was designed to monitor the safety and adverse reactions of the injection in clinical use. The study involved 12 hospitals and has not yet been officially published (Table 1).

**TABLE 1:** Details of our search on the clinical trial website (http://clinicaltrial.gov/) with keywords “*Erigeron breviscapus*” and “Dengzhan”.

NCT number	Start date	Study type	Recruitment status	Condition/disease	Intervention/treatment	Title
NCT00351806	July 2005	Interventional (clinical trial)	Completed	Cerebral infarction	*E. breviscapus* injection	AISTCM-The pathological pattern differentiation and outcome measurement of acute ischemic stroke treated with traditional chinese medicine
NCT00548223	December 2007	Interventional (clinical trial)	Completed	Stroke	Dengzhan shengmai capsule	Model study on the comprehensive treating protocol and effect evaluation of ischemic stroke with traditional chinese medicine
NCT00547950	November 2007	Interventional (clinical trial)	Unknown	Ischemic stroke	Deng Zhan Sheng Mai capsule	A model study on the comprehensive treating protocol of secondary prevention and effect evaluation of ischemic stroke with traditional chinese medicine
NCT02559960	September 2015	Observational	Suspended	Adverse drug event/reaction	Breviscapine powder injection	Post-marketing safety surveillance of breviscapine powder-injection: a registry study

## Anti-Ad Effects of *Erigeron breviscapus* (Vant.) Hand-Mazz

Previous studies have confirmed the therapeutic effects of EBHM in AD. For example, Huang et al. (2021) reported that DZSM capsules can improve cognitive function and the living capacity of patients with AD and suggested that inhibiting the production of neurotoxic substances (NO and ET) may be an anti-AD mechanism (Huang et al., 2021). Pu and Su (2020) also pointed out that EBHM and its active ingredients (scutellarin and CQA) may improve the learning and memory ability of AD animal models, and that its mechanisms may be associated with inhibition of Aβ aggregation, regulation of the cholinergic nervous system, inhibition of oxidative stress and inflammation, alleviation of tau hyperphosphorylation, and resistance to neuronal apoptosis (Pu and Su, 2020). However, research on the exact mechanism of EBHM and its active components in the treatment of AD is still lacking. Next, we elaborate on the anti-AD effects of EBHM and its active ingredients through different pathogenic mechanisms of AD (Table 2) to comprehensively summarize and analyze the pharmacological mechanism of EBHM in the treatment of AD and provide clues and a basis for subsequent studies of new therapeutic strategies for AD.

**TABLE 2:** The pharmacology and possible mechanisms of compounds and metabolites in *E. breviscapus* (Vant.) Hand-Mazz. for Alzheimer’s disease.

Active ingredients	Model	Administration	Pharmacological actions	Test index	Possible mechanism	References
Scutellarin	APP/PS1 mice	50 mg/kg, i.v.	Reduces soluble human Aβ42 and Aβ40 levels in the cortex	EPM, MWM	Targeting Aβ	Zhang et al. (2020)
Scutellarin	APP/PS1 mice	50 mg/kg, p.o.	Reduces Aβ in the brain and plasma, decreases pro-inflammatory cytokine expression	MWM	Targeting Aβ, neuroinflammation	Zeng et al. (2018)
Scutellarin	pBCAO rats	30 mg/kg, p.o.	Reduces Aβ formation by inhibiting APP and BACE-1 expression, inhibits the activation of glial cells	MWM	Targeting Aβ, neuroinflammation	Shin et al. (2018)
Scutellarin	Male wistar rats (Aβ ICV)	5 mg/ml, i.v.	Upregulates nAChR protein levels and AChE and BuChE activity	MWM	Targeting cholinergic neurotransmitter	Guo et al. (2011b)
Scutellarin	Wistar rats (Aβ_25-35_ ICV)	5 mg/ml, i.g.	Increases SOD and MAO levels, reduces IL-1, IL-6, TNF-α expression, and apoptotic neurons	MWM	Targeting oxidative stress, anti-apoptosis, neuroinflammation	Guo et al. (2013)
Scutellarin	Balb/c male mice (D-gal, AlCl3)	20 mg/kg, p.o.	Decreases p-tau and Aβ42 levels, enhances acetylcholine and SOD levels	MWM	Targeting tau protein, Aβ, oxidative stress	Hu et al. (2018)
Scutellarin	Wistar rats (Aβ_25-35_ ICV)	1 mg/ml, i.g.	Increases SOD activity, decreases MDA activity	MWM	Targeting oxidative stress, anti-apoptosis	Guo et al. (2011a)
Scutellarein/scutellarin	Male wistar rats (Aβ injected frontal cortex)	50 mg/kg, i.p./i.g.	Increases the ratio of cytoplasmic/nuclear NF-κB	MWM	Targeting neuroinflammation, anti-apoptosis	Huang et al. (2019)
Scutellarin–rivastigmine hybrids	Kunming mice (scopolamine)	2/4/8 mg/kg, i.g.	Decreases AChE vitality, increases ChAT vitality	Y-maze test	Targeting cholinergic neurotransmitter	Sang et al. (2015)
Baicalin	Male ICR mice (Aβ-injected hippocampus)	100 mg/kg, i.g.	Attenuates glial cell activation, decreases inflammatory factor (IL-6, TNF-α) expressions	MWM, probe test	Targeting neuroinflammation	Chen et al. (2015)
Baicalin	APP/PS1 mice	100 mg/kg, i.p.	Decreases the number of activated microglia and the level of proinflammatory cytokines	MWM, probe test	Targeting neuroinflammation	Jin et al. (2019)
Baicalin	PS1/APPsw mice	100 mg/kg, i.p.	Inhibits microglial activation, reduces inflammatory cytokine secretion	—	Targeting neuroinflammation	Xiong et al. (2014)
Baicalin	SH-SY5Y cells	100 μM	Inhibits Aβ1-42 aggregation, decreases H2O2 production	—	Targeting Aβ, neuroinflammation	Yin et al. (2011)
Baicalin	N2a/APPswe cells	1/5/10 μmol/L	Increases SOD activity, inhibits MDA production	—	Targeting oxidative stress	Cao et al. (2015)
Baicalin	Wistar rats (Aβ-injected hippocampus)	50/100/200 mg/kg, i.p.	Restores antioxidant enzyme activity, increases Bax/Bcl-2 ratio, caspase-9/-3 activation, and cytochrome c release	MWM	Targeting oxidative stress, anti-apoptosis	Ding et al. (2015)
Baicalin	Wistar rats (Aβ-injected hippocampus)	40 mg/kg, i.p.	Decreases hippocampal cyclooxygenase expression	T-Morris tests	Targeting anti-apoptosis	Li et al. (2011)
Baicalin	Male C57 mice (AβO)-induced	30/60 mg/kg, i.g.	Improves synaptic plasticity and mitochondrial fragmentation, rescues dysfunction	Y-maze tests	Targeting mitochondrial dysfunction	Yu et al. (2022)
5-CQA	APP/PS2 transgenic mice	0.8% (w/w)	Modulates Aβ and neuronal loss	Y-maze, novel object recognition	Targeting Aβ	Ishida et al. (2020)
4, 5-di-CQA/TCQA	SH-SY5Y cells	1/10/20 μM	Inhibits the aggregation of Aβ42	—	Targeting Aβ	Miyamae et al. (2012)
5-CQA	SH-SY5Y cells	50/25/12.5/6.25 μM	Reduces the apoptosis rate, promotes autophagic cellular degradation, increases autophagic flux	—	Targeting oxidative stress, anti-apoptosis	Gao et al. (2021)
3,5-di-O-CQA	SAMP mice	6.7 mg/kg	Upregulates PGK1 expression, activates ATP production	MWM	Targeting ATP production	Han et al. (2010)
TCQA	SAMP mice	5 mg/kg	Increases neurogenesis of the hippocampal dentate gyrus and proliferation of neural progenitor cells	MWM	Targeting hippocampal neurogenesis	Sasaki et al. (2019)

TCQA, 3,4,5-tricaffeoylquinic acid; 5-CQA, 5-caffeoylquinic acid; Aβ, amyloid β-peptide; AβO, Aβ oligomer; AChE, acetylcholinesterase; ATP, adenosine triphosphate; ChAT, acetyltransferase; BuChE, butyrylcholinesterase; EPM, elevated plus maze; ICV, intracerebroventricular injection; i.p., intraperitoneal; i.v., injection; MDA, malondialdehyde; MWM, morris water maze; MTT, 3-(4,5)-dimethylthiadiazo (-z-y1)-3,5-di-phenyltetrazolium romideopen; nAChRs, nicotinic acetylcholine receptors; p.o., persral; PGK1, phosphoglycerate kinase-1; SAMP, senescence-accelerated-prone; SOD, superoxide dismutase.

### Targeting Amyloid β-peptide

Amyloid β-peptide (Aβ) is a small molecular fragment formed by the two-step proteolytic hydrolysis of β-secretase and γ-secretase under the regulation of amyloid precursor protein (APP), which exists in the form of monomers, dimers, polymers, and fiber polymers, such as Aβ40 and Aβ42 (Lane et al., 2018). Increased Aβ production, increased Aβ42/Aβ40 ratio, promotion of Aβ deposition, and reduction of Aβ clearance result in the formation and deposition of SPs, followed by a complex cascade of inflammatory responses, microglial activation, and cytokine release (Selkoe and Hardy, 2016; Panza et al., 2019; Tiwari et al., 2019). These responses lead to progressive neurogenic damage, neuronal defects, and cognitive dysfunctions. Therefore, reducing Aβ production and removing Aβ deposition may be important therapeutic strategies in the treatment of AD.

Scutellarin (5, 6, 4’-trihydroxyflavone-7-glucuronide) has been identified as a major active ingredient of EBHM and has multiple pharmacological activities, including anti-inflammatory, anti-apoptosis, and anti-oxidation (Wang and Ma, 2018; Yang et al., 2018). Scutellarin has been reported to inhibit the aggregation of Aβ *in vitro* and prevent Aβ-mediated cell death when applied to cultured neuronal PC12 cells (Zhu et al., 2009). In animal models of AD, scutellarin reduced cognitive dysfunction in APP/PS1 mice, inhibited amyloid deposition, and reduced soluble Aβ42 and Aβ40 levels in the cortex of mice. In *in vitro* experiments, scutellarin prevented cognitive decline by converting Aβ monomers into low-toxicity amyloid fibrils or protofibrils, and by reducing the levels of highly toxic soluble Aβ oligomers (Zhang et al., 2020). Zeng et al. (2018) further confirmed that continuous administration of scutellarin significantly reduced the latency to find a platform and improved swimming in APP/PS1 mice. In addition, scutellarin reduced the levels of soluble and insoluble Aβ in the brain and plasma of mice; decreased the levels of Aβ plaque-associated gliosis, pro-inflammatory cytokines, tumor necrosis factor (TNF)-α, and interleukin (IL)-6; alleviated neuroinflammation; and showed anti-amyloidosis effects (Zeng et al., 2018). In a rat model of chronic cerebral hypoperfusion, scutellarin exhibited an anti-Aβ effect. Treatment with 30 mg/kg scutellarin for 4 weeks significantly ameliorated spatial cognitive impairment and memory deficits in rats with permanent bilateral common carotid artery occlusion (pBCAO). Further experiments showed that scutellarin reduced Aβ formation by inhibiting the expression of APP and β-site APP cleaving enzyme 1 (BACE-1) in the hippocampi of pBCAO rats. Additionally, scutellarin (30 mg/kg) significantly suppressed the expression of glial fibrillary acidic protein and Iba1 in the cerebral cortex and hippocampal tissue, thereby inhibiting the activation of glial cells (microglia and astrocytes) in brain tissues (Shin et al., 2018).

Previous studies have suggested that CQA can improve or enhance the learning and memory abilities of various animal models of AD. For instance, 5-O-caffeoylquinic acid (5-CQA) supplementation significantly reduces hippocampal Aβ plaque formation and neuronal loss. This neuroprotective effect is due to 5-CQA upregulation of the gene encoding low-density lipoprotein receptor-related protein 1 (Aβ efflux receptor) and normalization of the perivascular localization of aquaporin 4, thereby promoting Aβ clearance along paravascular pathways (Ishida et al., 2020). In addition, in the SH-SY5Y cell model, CQA exhibited anti-amyloidogenic properties. 4, 5-di-CQA and 3, 4, 5-tri-CQA could suppress β-sheet transformation and cytotoxicity in SH-SY5Y of Aβ42 in a dose-dependent manner. Furthermore, 3, 4, 5-tri-CQA blocked the formation of Aβ42 oligomers, indicating that 3, 4, 5-tri-CQA may be a potential agent for the prevention of AD (Miyamae et al., 2012).

### Targeting Tau Proteins

Tau is a multifunctional protein related to microtubule function, and the stability of the microtubule structure in neurons is conducive to the transfer of nutrients or information molecules between synapses (Stancu et al., 2019). However, in patients with AD, tau does not play a role in promoting microtubule assembly and enhancing microtubule stability. Instead, it separates from microtubules, attaches to other Tau molecules, self-assembles into pairs of helices, and reassembles into NFTs (Gao et al., 2018). The main component of NFTs is the hyperphosphorylated tau (p-Tau) protein, which is neurotoxic and destroys the structure of neurons, leading to abnormal communication and signal processing between neurons and neuronal apoptosis (Gibbons et al., 2019). This suggests that using tau protein as an important target and preventing its spread and accumulation may alleviate the progression of AD disease (Jouanne et al., 2017).

In an aluminum chloride plus D-galactose-induced AD mouse model, scutellarin enhanced horizontal and vertical movements in an autonomic activity test and reduced the escape latency time of mice in the Morris water maze (MWM) test. Scutellarin administration significantly reduced the levels of p-Tau, which indicated that the attenuation of the effects associated with AD following treatment with scutellarin may be due to the resultant decreased levels of p-Tau. In addition, the enhanced levels of acetylcholine and SOD in serum and brain lysates may also contribute to the protective effects of scutellarin (Hu et al., 2018).

### Targeting Cholinergic Neurotransmitter

Acetylcholine (ACh) is primarily involved in arousal, learning, memory, and motor regulation. ACh is widely distributed throughout the cortex, basal ganglia, and basal forebrain, suggesting that cholinergic transmission is essential for brain function (Hampel et al., 2018). The degree of cognitive dysfunction in patients with AD is closely related to a decrease in acetylcholinesterase (AChE) activity and ACh synthesis (Ferreira-Vieira et al., 2016). The cholinergic hypothesis suggests that cholinergic neurons are affected in the early stage of AD and that the degeneration of cholinergic neurons in the basal forebrain, the reduction of cholinergic neurotransmitters in the cerebral cortex and other regions, and the loss of cholinergic agents involved in ACh synthesis are the main causes of cognitive decline in patients with AD (Machado et al., 2020). Therefore, increasing ACh synaptic levels or selective agonists modulating acetylcholine receptors (AChRs) in the postsynaptic membrane can increase the continuous accumulation of ACh and AChR activation in nerve cells, thereby reversing cognitive dysfunction (Gray et al., 2015; Sultzer, 2018).

Scutellarin also showed its ability to improve learning and memory defects in an Aβ-induced rat dementia model, which was attributed to scutellarin treatment, which significantly upregulated the α4 and α7 AChR subunit protein levels by 24% and 30%, respectively. Moreover, maintenance of AChE and butyrylcholinesterase activities in the brain and plasma of Aβ-induced deficits is also an important therapeutic mechanism of scutellarin (Guo et al., 2011b). A scutellarin–rivastigmine hybrid (compound 15c) had a neuroprotective effect against H2O2-induced PC12 cell injury and could cross the blood–brain barrier *in vitro*. Moreover, compound 15c also had significant neuroprotective effects in scopolamine-induced cognitive impairment in mice and could decrease the vitality of AChE and increase the vitality of acetyltransferase in the hippocampus of mice (Sang et al., 2015).

### Targeting Neuroinflammation

With the deepening of the study on AD pathogenesis, neuroinflammatory mechanisms are gradually being recognized to be involved in the pathogenesis and development of AD (Leng and Edison, 2021). Microglia (MG) are the most critical mediators of immune response in the brain. MG in the resting state functions in migration and swallowing and plays a main role in nutrition and support. However, it can be activated into M1 (pro-inflammatory activation) and M2 (anti-inflammatory activation) activation states under the stimulation of internal and external environmental factors (Tang and Le, 2016). Studies have shown that M1 type MG in patients with AD oversecretes proinflammatory factors, such as TNF-α, INF-γ, IL-1 β, IL-6, and other inflammatory cytokines involved in neurotoxicity and neuronal death (Morales et al., 2014). Under physiological conditions, astrocytes are evenly distributed and can secrete neurotransmitters and neurotrophic factors to participate in the maintenance of the normal neuronal microenvironment, signal transduction, immune regulation, and other functional activities (Sofroniew and Vinters, 2010). In the AD brain, abnormal accumulation of astrocytes around amyloid plaques has been observed, indicating that astrocytes have a scavenging effect on Aβ deposition (Rodríguez-Arellano et al., 2016). In mouse models of AD, decreased astrocyte activity has been found, which may be related to increased Aβ deposition and significantly increased clearance load (Kraft et al., 2013). Therefore, regulating the inflammatory state of MG and the function and activity of astrocytes may be potential targets in the treatment of AD (Fakhoury, 2018; Du et al., 2021).

Nuclear factor of activated B-cells (NF-κB) is a protein complex that controls DNA transcription and can be found in almost all common animal cell types (Stormberg et al., 2021). It plays an important role in regulating the immune response, and is implicated in cellular responses to stimuli such as cytokines, free radicals and stress (Ju Hwang et al., 2019). NF-κB activation, as an underlying cause of AD, was found predominantly in neurons and glial cells in Aβ plaque surrounding areas (Yang et al., 2021). In addition, there was a strong correlation between increased NF-κB activity and cyclooxygenase-2 (COX-2) transcription in the superior temporal lobe gyrus of AD patients was also demonstrated (Wang et al., 2014). Moreover, the levels of NF-κB activity were increased in cholinergic neurons in the basal forebrains of AD patients (Li et al., 2019), and could affect AD pathology *via* increased pro-inflammatory cytokines expression and amyloidogenesis (Xie et al., 2020). Therefore, inhibiting NF-κB are predicted to play a protective role in the development of AD (Seo et al., 2018). Scutellarein (5, 6, 7, 4′-tetrahydroxy flavone) is the hydrolyzed product of scutellarin in perennial herbs. A previous study demonstrated that scutellarein is absorbed more easily following oral administration than is scutellarin (Mamadalieva et al., 2011). In Aβ-treated PC 12 cells, pretreatment with scutellarein and scutellarin increased cell viability, significantly attenuated Aβ-induced cell death, and decreased apoptotic ratios. Furthermore, scutellarein and scutellarin increased the expression level of cytoplasmic NF-κB and decreased the expression level of nuclear NF-κB, suggesting that scutellarein and scutellarin inhibit the NF-κB signaling pathway. In an *in vivo* study, scutellarein and scutellarin significantly improved the Aβ-induced latency to locate the platform during the acquisition period and increased the number of platform crossings in a spatial probe trial. Scutellarein and scutellarin restored the decreased B-cell lymphoma 2 (Bcl-2) and increased Bax and cleaved caspase-3 levels in the hippocampus of Aβ-induced rats, suggesting their anti-apoptotic effects, although scutellarein had more prominent effects than scutellarin. Scutellarein also showed anti-neuroinflammatory activity, significantly increased cytoplasmic NF-κB and decreased nuclear NF-κB compared to the Aβ group. Thus, scutellarein may inhibit the activation of NF-κB signaling in the hippocampus (Huang et al., 2019).

Baicalin is another major active flavonoid extracted from EBHM and has been shown to have anti-inflammatory and anti-tumor effects (Wu et al., 2013). In an Aβ1-42 induced mice model, a 14-day administration of baicalin (100 mg/kg) significantly ameliorated memory impairment in the MWM and probe tests, attenuated glial cell activation, and decreased the expression of inflammatory factors (IL-6 and TNF-α), suggesting that baicalin ameliorated Aβ1-42 protein-related pathology and cognitive dysfunction *via* its anti-neuroinflammatory activity (Chen et al., 2015). Furthermore, baicalin administration effectively decreased the number of activated MGs and proinflammatory cytokine levels (IL-1β, IL-18, and iNOS), as well as neuroinflammation-mediated neuronal apoptosis *via* inhibition of the activation of NLR family pyrin domain containing 3 (NLRP3) inflammasomes and toll-like receptor 4 (TLR4)/NF-κB signaling pathway, and attenuated spatial memory dysfunction in APP/PS1 mice (Jin et al., 2019). In another AD transgenic mouse model, baicalin inhibited Aβ-induced microglial activation and reduced Aβ-induced inflammatory cytokine (IL-6, TNF-α, and NO) secretion *via* the Janus kinase 2/signal transducer and activator of transcription 3 (JAK2/STAT3) signaling pathway, thus providing a new means for the prevention and treatment of AD (Xiong et al., 2014).

### Targeting Oxidative Stress

Oxidative stress (OS) refers to a pathological state in which free radicals or other products in the body exceed the body’s antioxidant capacity (Chang et al., 2020). Excessive ROS and reactive nitrogen species (RNS) are produced (Smallwood et al., 2018). Under pathological conditions, a variety of cells, such as cortical neurons and astrocytes, can produce excessive NO, which reacts with superoxide anions to produce more active peroxynitry (ONOO-) and·OH, thus causing potential damage to the body. The OS response is characterized by the production of ROS and RNS and an imbalance in antioxidant defense (Chiarini et al., 2016), which are closely involved in the pathogenesis of AD (Ahmad et al., 2017). Antioxidants are generally divided into enzymatic and nonenzymatic systems. The former includes catalase, SOD, and glutathione peroxidase (GSH-PX). The latter includes vitamins, amino acids, and metalloproteins (Margaritelis et al., 2018). By scavenging free radicals and/or decomposing hydrogen peroxide, the oxidation chain is blocked, which delays the occurrence and development of AD (Khan et al., 2019).

In Aβ25-35 induced AD rats, scutellarin shortened the latent escape period and the time required to pass the original site of the platform, and increased the number of crossings. Scutellarin reversed the reduced activity of SOD and elevated levels of monoamine oxidase (MAO), decreased the upregulation of IL-1, IL-6, and TNF-α in the cortex, and reduced the percentage of apoptotic neurons in the rat brain (Guo et al., 2013). In another group of Aβ25-35 AD-induced rats, similar conclusions were obtained; that is, the application of scutellarin significantly increased SOD activity and decreased MDA activity in brain tissues for 30 consecutive days, and scutellarin played an anti-apoptotic role in the brain tissues of rats (Guo et al., 2011a).

Baicalin may also be a potential anti-OS agent. In an *in vitro* study, baicalin prevented SH-SY5Y cells against damage by directly interacting with copper to inhibit Aβ1-42 aggregation. In addition, it protected SH-SY5Y cells from oxidative injuries induced by Aβ1-42 aggregation by decreasing H2O2 production (Yin et al., 2011). In the N2a/APPswe cell line, pretreatment with baicalin improved cell viability by increasing SOD activity, inhibiting MDA production, reducing nuclear NF-κB protein levels, and promoting nuclear factor erythroid 2-related factor 2 (NRF2) translocation to the nucleus, indicating that baicalin has anti-oxidative stress effects (Cao et al., 2015).

CQA has various pharmacological properties, including antioxidant activity. For example, 5-CQA reduced the rate of apoptosis, increased cell viability, and improved cell morphology in H2O2-treated SH-SY5Y cells. In addition, 5-CQA alleviated the accumulation of autophagic vacuoles and decreased P62 levels and the LC3B II/I ratio, thereby promoting autophagic cellular degradation and increasing autophagic flux (Gao et al., 2021).

### Targeting Anti-Apoptosis

Apoptosis refers to programmed cell death that occurs during cell development or under the action of certain factors through the regulation of genes and their products in cells. Neuronal apoptosis plays an important role in neurodegenerative diseases, such as AD, which can lead to the loss of a large number of neurons (Bredesen, 1995; Caccamo et al., 2017). It is influenced by Aβ, Bcl-2-associated X protein (Bax), Bcl2, caspases, TNF-α, ROS, and enzyme perturbation (Dhivya Bharathi et al., 2019). The mechanisms of neuronal apoptosis include the following: 1) the extrinsic pathway, initiated by cell surface death receptors such as Fas and tumor necrosis factor receptor families; 2) the intrinsic pathway or mitochondrial pathway, initiated by stress conditions, chemotherapeutic agents, and drugs; and 3) activation of caspase 12 caused by endoplasmic reticulum stress, leading to apoptosis (Boatright and Salvesen, 2003). Drugs with anti-apoptotic activity and potential applications in targeting apoptosis in AD include flavonoids and antioxidants of plants (Obulesu and Lakshmi, 2014).

Considering the involvement of Aβ-induced OS in the etiology and pathology of AD, antioxidant therapy to scavenge excess ROS by inducing endogenous antioxidant enzymes is a promising approach to the prevention of AD (Park, 2010). Ding et al. (2015) suggested that baicalin has beneficial effects on learning and memory deficits caused by Aβ in rats. The neuroprotective effects of baicalin may be achieved by its antioxidant and anti-apoptotic activities. Baicalin treatment improves antioxidant capacity by restoring the activities of antioxidant enzymes (SOD, catalase, and GSH-PX) and upregulating their gene expression. In addition, baicalin can effectively prevent Aβ-induced reduction in mitochondrial membrane potential, an increase in the Bax/Bcl-2 ratio, activation of Caspase-9/-3, and the release of cytochrome c. Moreover, baicalin treatment significantly inhibits the inhibitory effect of Aβ on Nrf2 and exerts a stronger antioxidant effect (Ding et al., 2015). COX-2 is a key enzyme in neuronal death and is an important marker of the inflammatory response. The protein expression levels of COX-2 and its upstream gene peroxisome proliferator-activated receptor γ (PPAR γ) is increased in patients with AD and is positively correlated with the level of Aβ. COX-2 inhibitors can slow the pathological evolution of AD by inhibiting COX (Pasinetti, 2002). In a study by Li et al. (2011), baicalin inhibited hippocampal COX-2 expression, antagonized Aβ-induced neuronal apoptosis, and diminished cortical and hippocampal neuron necrosis.

### Other Possible Mechanisms

EBHM and its active ingredients may play a therapeutic role in AD *via* other neuroprotective mechanisms. Baicalin plays a significant role in rescuing mitochondrial dysfunction by modulating mitochondrial fragmentation. Yu et al. (2022) reported that baicalin treatment significantly reversed the altered learning and memory behaviors of an Aβ oligomer-induced mouse model due to improved mitochondrial fragmentation, synaptic plasticity, and rescue of dysfunction *via* inhibition of phosphodiesterase-4, leading to activation of the phosphorylated Ser637 site of mitochondrial dynamin-related protein 1. In senescence-accelerated prone (SAMP) mice, 3, 5-di-O-CQA administration improved spatial learning and memory *via* upregulation of phosphoglycerate kinase-1 expression and activation of ATP production (Han et al., 2010). 3, 4, 5-tri-CQA exhibited anti-AD effects in the same AD mouse model (SAMP). Treatment with 3, 4, 5-tri-CQA improved spatial learning and memory by increasing neurogenesis in the hippocampal dentate gyrus and by pro-neurogenic effects in human cells, which might have occurred *via* activation of the bone morphogenetic protein signaling pathway. Moreover, 3, 4, 5-tri-CQA may be a new agent capable of increasing neural progenitor cell proliferation in the dentate gyrus, suggesting that 3, 4, 5-tri-CQA may be a new therapeutic drug for treating AD (Sasaki et al., 2019).

## Conclusion

**FIGURE 3:**
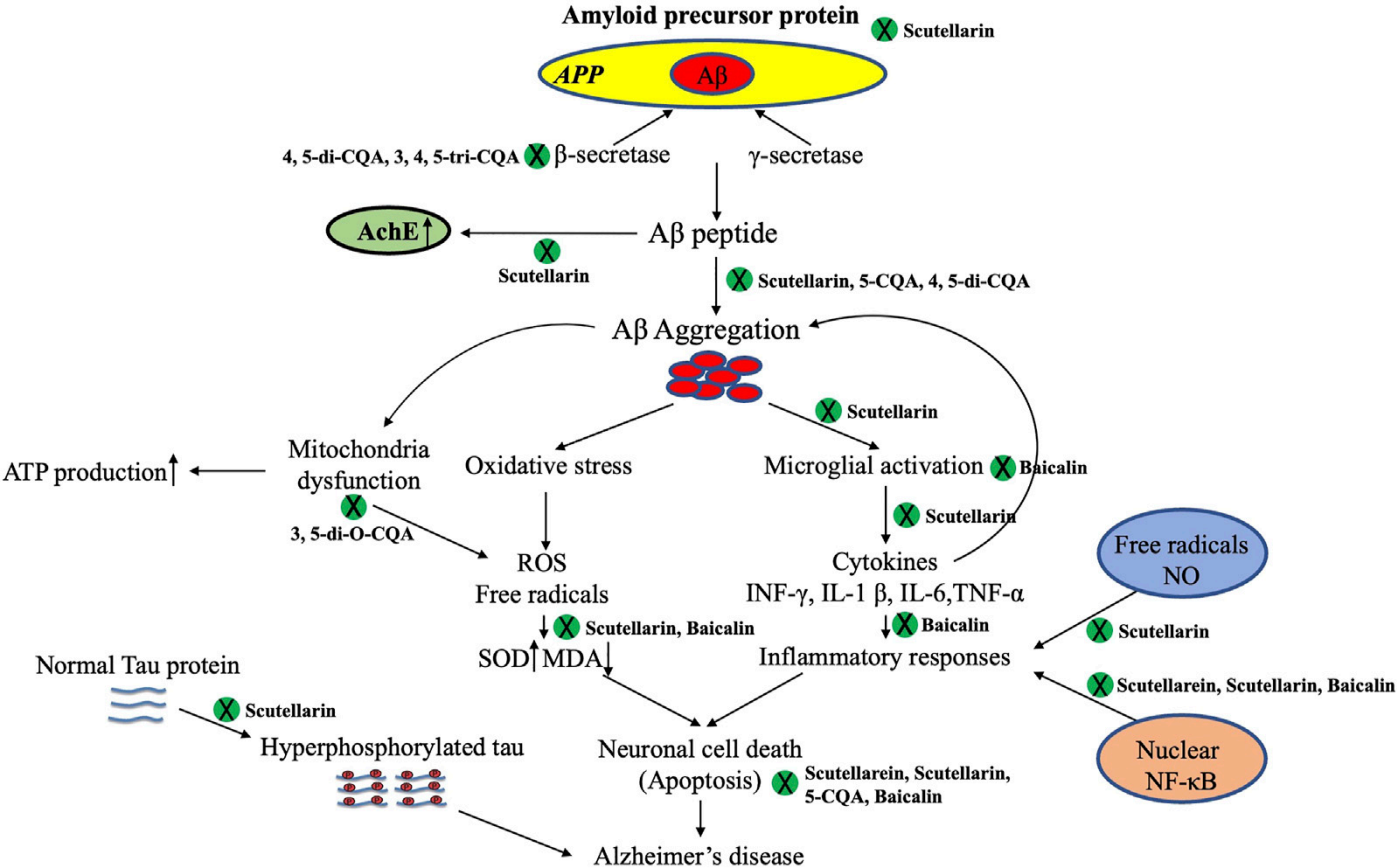
Different active constituents of *Erigeron breviscapus* (Vant.) Hand-Mazz. have shown anti-AD effects against Aβ deposition, hyperphosphorylated Tau protein, cholinergic neurotransmitters, neural apoptosis, oxidative stress, and neuroinflammation. 5-CQA, 5-O-caffeoylquinic acid; 4, 5-di-CQA, 4, 5-dicaffeoylquinic acid; 3, 4, 5-tri-CQA, 3, 4, 5-tricaffeoylquinic acid; AD, Alzheimer’s Disease; ATP, adenosine triphosphate; MDA, malondialdehyde; ROS, reactive oxygen species; SOD, superoxide dismutase.

Based on current research, pathological features of AD mainly include Aβ oligomers, tau protein hyperphosphorylation, Aβ plaque deposition, disordered brain tissue energy metabolism, brain neuron apoptosis, inflammatory response, and OS injury. As the etiology of AD has not been clearly elucidated, it is difficult to develop effective drugs to prevent and treat AD. Drugs that reduce Aβ levels, including γ-secretase inhibitors/modulators, BACE-1 inhibitors that reduce Aβ production, and active/passive immune agents that prevent Aβ aggregation or promote Aβ clearance (e.g., antibodies to Aβ protein), did not improve cognitive function in patients with AD in clinical trials (Huang et al., 2020). Therefore, it is speculated that treatment of AD with a single target may not achieve the desired effect. Hence, the future direction of AD drug research is based on a multitarget design. TCM has great potential for treating AD because of the synergistic effects of multiple components, multiple pathways, and multiple targets. Many TCMs, including EBHM, have obvious advantages in improving cognitive dysfunction and require further study. Current studies have shown that scutellarin, baicalin, and CQA, the main components of EBHM, have good pharmacological effects and research prospects in treating cognitive dysfunction. Its mechanisms include reducing Aβ toxicity, inhibiting tau phosphorylation, providing an anti-inflammatory response, regulating the central cholinergic system, resisting OS, providing anti-apoptosis activity, and promoting nerve cell proliferation and differentiation (Figure 3).

However, there is still a long way to go before EBHM can be used as a clinical drug for treating AD. First, anti-AD studies on the active ingredients of EBHM, including scutellarin, are mainly focused on animal models and have not been applied in clinical controlled studies on patients with AD. Second, there is a lack of in-depth research on the synergistic relationship between the pharmacological mechanisms of the active components of EBHM and AD. For example, different proportions of CQA components exhibit different activities. Some CQA components have no significant anti-AD activity, whereas others have a synergistic effect after combination (Oboh et al., 2013). Finally, although there are many studies on animal models of AD, the dosages of EBHM and its active ingredients are often different. Hence, the standardized drug intervention dose, administration time, and pharmacokinetic studies require further improvements. Nevertheless, elucidation of the structure of EBHM’s active ingredients and progress in pharmacological mechanisms provides a promising approach for the treatment of AD.
